# Anchorage of Au^3+^ into Modified Isoreticular Metal–Organic Framework-3 as a Heterogeneous Catalyst for the Synthesis of Propargylamines

**DOI:** 10.1038/s41598-017-13081-0

**Published:** 2017-10-05

**Authors:** Lili Liu, Xishi Tai, Xiaojing Zhou, Chunling Xin, Yongmei Yan

**Affiliations:** School of Chemistry & Chemical Engineering and Environmental Engineering, Weifang University, Weifang, 261061 China

## Abstract

Postsynthetic modification of metal-organic framework is a general and practical approach to access MOF-based catalysts bearing multiple active sites. The isoreticular metal–organic framework-3 (IRMOF-3) was modified with lactic acid through condensation reaction of the carboxyl group of lactic acid and amino group present in IRMOF-3 frameworks. Au^3+^ was subsequently anchored onto the metal–organic framework IRMOF-3 using postsynthetic modification. The synthezized IRMOF-3-LA-Au (LA = lactic acid) was characterized by powder X-ray diffraction, N_2_ adsorption-desorption, infrared spectroscopy, liquid-state nuclear magnetic resonance, thermogravimetric analysis, H_2_-temperature programmed reduction, transmission electro microscopy, and inductively coupled plasma–optical emission spectrometry. IRMOF-3-LA-Au acted as an efficient heterogeneous catalyst in the synthesis of propargylamines by three-component coupling reaction of aldehyde, alkyne, and amine. Moreover, the catalyst is applicable to various substituted substrates, including aromatic and aliphatic aldehydes, alkyl- and aryl-substituted terminal alkynes, and alicyclic amines. In addition, the catalyst can be easily separated from the mixture and can be reused for four consecutive cycles.

## Introduction

The design and synthesis of novel molecular scaffolds with unique structural and biological properties is an interesting challenge^1,2^. Propargylamines exhibit biological properties, such as anti-Alzheimer, anti-Parkinsonian, and antidepressant activities^3–5^. Methods used to prepare propargylamines include amination of propargylic electrophiles^6^, nucleophilic addition of alkynes to imines or enamines^7^, and three-component (aldehyde, terminal alkyne, and amine, A^3^) coupling reactions through C–H activation^8,9^. Among these synthetic strategies, A^3^ coupling is the most efficient, and it permits construction of complex structures from simple starting materials with water as byproduct^10,11^. In recent years, various metals such as Au^9^, Cu^8^, Ag^12^, In^13^, Fe^14^, Zn^15^, and Ni^16^ were extensively used to synthesize propargylamines. Among these metals, cationic gold species have received special attention because of their effective catalytic activity in A^3^ coupling reactions^17,18^. Development of efficient, environmentally friendly, and economical catalysts to synthesis of propargylamines has become more important in green and sustainable development in the chemical industry. Homogeneous catalysts have many attractive properties, such as high activities and selectivities. However, many homogeneous catalytic systems cannot be commercialized because of difficulties associated with separating the products from the catalyst^19^. Heterogeneous catalysts often have lower activity or selectivity but can be easily reused. The recent researches had shown that metal–organic frameworks (MOFs) could bridge the gap between homogeneous and heterogeneous catalysis^20,21^.

MOFs are a relatively new class of inorganic–organic hybrid materials, which have tremendously attracted interest due to their potential applications in gas storage and adsorption^22,23^, molecular separation^24,25^, fluorescence^26,27^, and sensors^28,29^. Moreover, MOFs have aroused interest in heterogeneous catalysis owing to their high surface areas, uniform pores, and chemical tunability^30,31^. Postsynthetic modification of MOFs has recently been employed as a general approach for incorporating a wide range of functional groups into MOFs^32–37^. Topologically diverse MOFs can be customized by postsynthetic modification with different functional groups^38–42^. Wittmann *et al*.^42^ synthesized the remarkable stabilisation of the mesoporous MOF Al-MIL-101-NH_2_ by postsynthetic modification with phenyl isocyanate. Moreover, UMCM-1-NH_2_ was modified with two metal-binding groups that were metallated with Fe^3+^ and Cu^2+^ to yield UMCM-1-AMFesal and UMCM-1-AMCupz, respectively^33^. UMCM-1-AMFesal is catalytically active in Mukaiyama–aldol reaction over multiple catalytic cycles without showing loss of activity or crystallinity^33^. Gee *et al*.^35^ prepared DMOF-1-NHak through postsynthetic modification using diketene. Candu *et al*.^43^ prepared a highly active and enantioselective MOF through postsynthetic modification of [Cu_2_(mand)_2_(hmt)] (mand = mandelic acid, hmt = hexamethylenetetramine) with chiral and dimeric Cr^3+^ salen complexes. Tabatabaeian *et al*.^44^ designed a novel heterogeneous catalyst for oxidation reactions by anchoring a Ru Schiff base moiety onto microporous isoreticular metal–organic framework-3 (IRMOF-3) via postsynthetic method. The catalyst shows high activity toward selective oxidation of arenes and heteroaromatic compounds and for oxidation of sulfids to sulfoxides and sulfones.

In the present study, we designed and synthesized a new Au^3+^–containing catalyst, IRMOF-3-LA-Au, through postsynthetic modification of IRMOF-3 with lactic acid and gold. IRMOF-3-LA-Au was found to be active and selective for catalyzing A^3^ coupling reaction of aldehyde, alkyne, and amine in 1,4-dioxane. They display better catalytic activity than the catalyst containing Au° nanoparticles. Furthermore, the catalyst is compatible with various substituted substrates and could be reused for at least four reaction cycles.

## Results and Discussion

### Synthesis and Characterization

**Figure 1:**
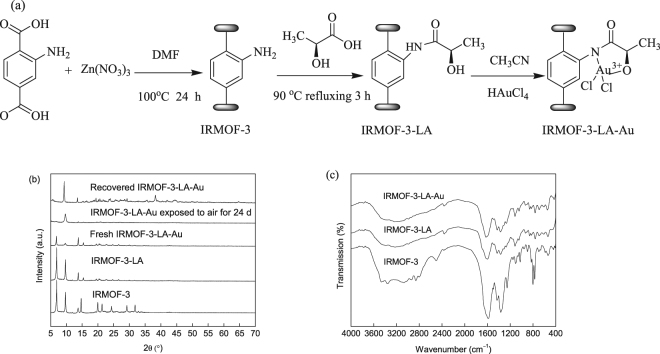
(**a**) Procedure for obtaining the Au^3+^-containing catalyst IRMOF-3-LA-Au, (**b**) X-ray diffraction patterns of IRMOF-3, RMOF-3-LA, IRMOF-3-LA-Au, and recovered IRMOF-3-LA-Au (**c**) IR spectrum of as-synthesized IRMOF-3, IRMOF-3-LA, and IRMOF-3-LA-Au.

The condensation of the amine-functionalized framework IRMOF-3 and salicylaldehyde has been reported in our previously study^17^. The present study investigated the ability of lactic acid to modify IRMOF-3 and generate a new functionalized MOF IRMOF-3-LA in a parallel approximation. The functionalized IRMOF-3-LA was treated with HAuCl_4_ to generate the Au^3+^-containing catalyst IRMOF-3-LA-Au. Figure 1a shows the synthesis pathway in the functionalized MOF system. IRMOF-3 was synthesized via a solvothermal method at 100 °C for 24 h by using 2-aminoterephthalic acid, Zn(NO_3_)_2_·6H_2_O, and *N,N*′-dimethylformamide (DMF). The as-synthesized IRMOF-3 was washed with DMF and CHCl_3_ prior to postsynthetic modification to remove the unreacted 2-aminoterephthalic acid. IRMOF-3-LA was obtained via treatment with lactic acid in a 1:3.5 ratio in refluxing CH_3_CN for 3 h through condensation reaction of the carboxyl group of lactic acid and amino group present in IRMOF-3 frameworks. As shown in Fig. 1a, hydroxyl groups were successfully grafted onto IRMOF-3, and amide groups formed. The hydroxyl and amide groups of the resulting IRMOF-3-LA were used as anchoring groups to stabilize the Au^3+^ ions. The functionalized IRMOF-3-LA was subsequently treated with HAuCl_4_ to generate the Au^3+^–containing IRMOF-3-LA-Au catalyst.

The XRD patterns of IRMOF-3, IRMOF-3-LA, and IRMOF-3-LA-Au are compared in Fig. 1b. The powder X-ray diffraction (PXRD) spectra of IRMOF-3 prepared by the solvothermal method matched well with those reported results^36,45^. The cubic lattice of IRMOF-3 is well retained even after modification with lactic acid and HAuCl_4_. During the course of these investigations, IRMOF-3-LA-Au was observed to decompose in air. The XRD pattern of the sample changed significantly after exposure to air for 24 day. The intensity of the peak at 2θ = 6.8° decreased severely in XRD pattern. Joseph *et al*. reported that the reflections in XRD pattern for IRMOF-3 decrease over time after exposing to ambient air for 4 days^46^. The change in XRD patterns can be attributed to atmospheric water^47,48^. The peak centered at 2θ = 6.8° disappeared for IRMOF-3-LA-Au after four runs, suggesting that the structure of IRMOF-3 decomposed during A^3^ coupling reaction. Notably, in the XRD pattern of recovered IRMOF-3-LA-Au, the Au(111), Au(200), and Au(220) peaks were observed at 2θ = 38.3°, 44.5°, and 64.7°, respectively (JCPDS 7440-57-5). IRMOF-3, IRMOF-3-LA-Au, and recovered IRMOF-3-LA-Au exhibited type I isotherms at 77 K (Figure S1). The nitrogen sorption study revealed that the BET surface area and pore volume of IRMOF-3 were 1212 m^2^/g and 0.46 cm^3^/g, respectively. After modification with lactic acid and gold, IRMOF-3-LA-Au showed a BET surface area of 687 m^2^/g with a pore volume of 0.38 cm^3^/g. The surface area and pore volume of IRMOF-3-LA-Au were smaller than those of IRMOF-3. This characteristic likely resulted from the presence of lactic acid and gold in the pores of the catalyst. Recovered IRMOF-3-LA-Au showed a severely decrease in the BET surface area (32 m^2^/g) and pore volume (0.01 cm^3^/g) as a result of the decomposition of IRMOF-3 during A^3^ coupling reaction. IR spectroscopy was used to characterize the functional groups in IRMOF-3, IRMOF-3-LA, and IRMOF-3-LA-Au. Bands at 3473 and 3356 cm^−1^ correspond with the asymmetric and symmetric stretching vibrations of –NH_2_ of the NH_2_–BDC ligand, which demonstrates that the amino groups are free in the as-synthesized IRMOF-3^17,49^. In addition, a peak can be observed at 800 cm^−1^, which was assigned to the wagging vibration of –NH_2_ of the NH_2_–BDC ligand. The IRMOF-3 also displayed bands at 1591, 1361, and 1257 cm^−1^, and these bands are correspond to the C=C in the benzene ring, C–N, and C–O vibration^50^. After modification with lactic acid and gold, noticeable changes were observed at 3500–3200 cm^−1^. The –NH_2_ bands at 3473 and 3356 cm^−1^ notably weakened in IRMOF-3-LA and IRMOF-3-LA-Au samples. Furthermore, the band at 800 cm^−1^, which is assigned to the wagging vibration of –NH_2_, was disappeared after reaction with lactic acid. This indicated that the carboxyl group of lactic acid had reacted with amino group present in the frameworks of IRMOF-3. ^1^H-NMR analysis was further used to determine the percentage of linker functionalization by lactic acid (Figure S2). ^1^H-NMR analysis indicated that 55% of the amine groups in the IRMOF-3 framework were converted into amides calculated by comparing the areas of the –NH_2_ between the modified and unmodified IRMOF-3. The thermal stability of IRMOF-3-LA and IRMOF-3-LA-Au was identical to that of IRMOF-3, with a major weight loss between 350 °C and 500 °C caused by decomposition of the organic linkers of the framework (Figure S3).

**Figure 2:**
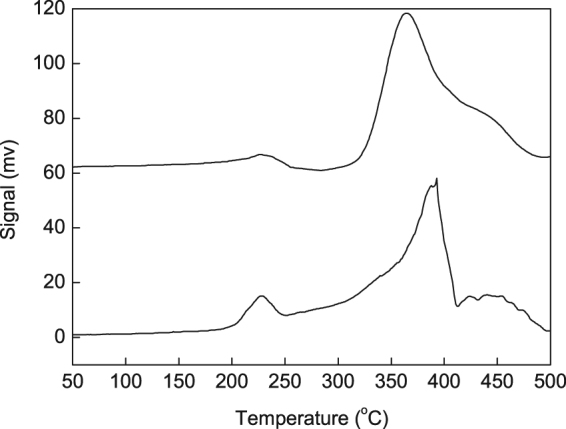
H_2_-TPR profiles characterizing the reduction temperature of Au^3+^ stabilized by metal-organic framework. (**a**) IRMOF-3-LA-Au (50 mg); (**b**) Recovered IRMOF-3-LA-Au after four cycles of A^3^ coupling reactions at 80 °C (50 mg).

Figure 2 presents the H_2_-TPR profiles of the as-synthesized IRMOF-3-LA-Au and the recovered IRMOF-3-LA-Au after four cycles of A^3^ coupling reaction at 80 °C in the examined temperature range (50 °C–500 °C). The TPR profiles of the as-synthesized IRMOF-3-LA-Au and recovered IRMOF-3-LA-Au exhibited two pronounced reduction peaks with the maximum in the range of 200 °C–250 °C and 300 °C–410 °C, respectively. We have reported that the catalyst of 4.6%Au/IRMOF-3 showed two reduction peaks in the range of 190 °C–270 °C and higher than 300 °C that was attributed to the reduction of Au^3+^ to Au° and the decomposition of MOF, respectively^17^. Zhang *et al*. reported that the reduction temperature of Au^3+^ ions in IRMOF-3-SI-Au is 239 °C, higher than that of Au^3+^ ions supported on nanocrystalline CeO_2_ (107 °C) or on ZrO_2_ (210 °C)^51–56^. Gil *et al*. reported that the TCD signals between 250 °C and 377 °C is mainly formed as consequence of the CO and CO_2_ release resulting from the decomposition of carboxylic groups^57^. Phan *et al*. reported that the peaks at 396 °C and 415 °C were attributed to the reduction of CuO structures formed during the total collapse of the Cu_2_(BDC)_2_(DABCO) structure^58^. Similar to a previous result, the H_2_-consumption peak observed at a low temperature range (200 °C–250 °C) is attributed to the reduction of Au^3+^ ions into Au° nanoparticles^51^. The higher temperature peak is attributed to the decomposition of IRMOF-3^51^. The much lower intensities of the TCD signals of the recovered IRMOF-3-LA-Au after four successive cycles in the range of 200 °C–250 °C than that for the as-synthesized IRMOF-3-LA-Au, suggesting a certain amount of Au^3+^ was reduced into Au° during A^3^ coupling reaction.

**Figure 3:**
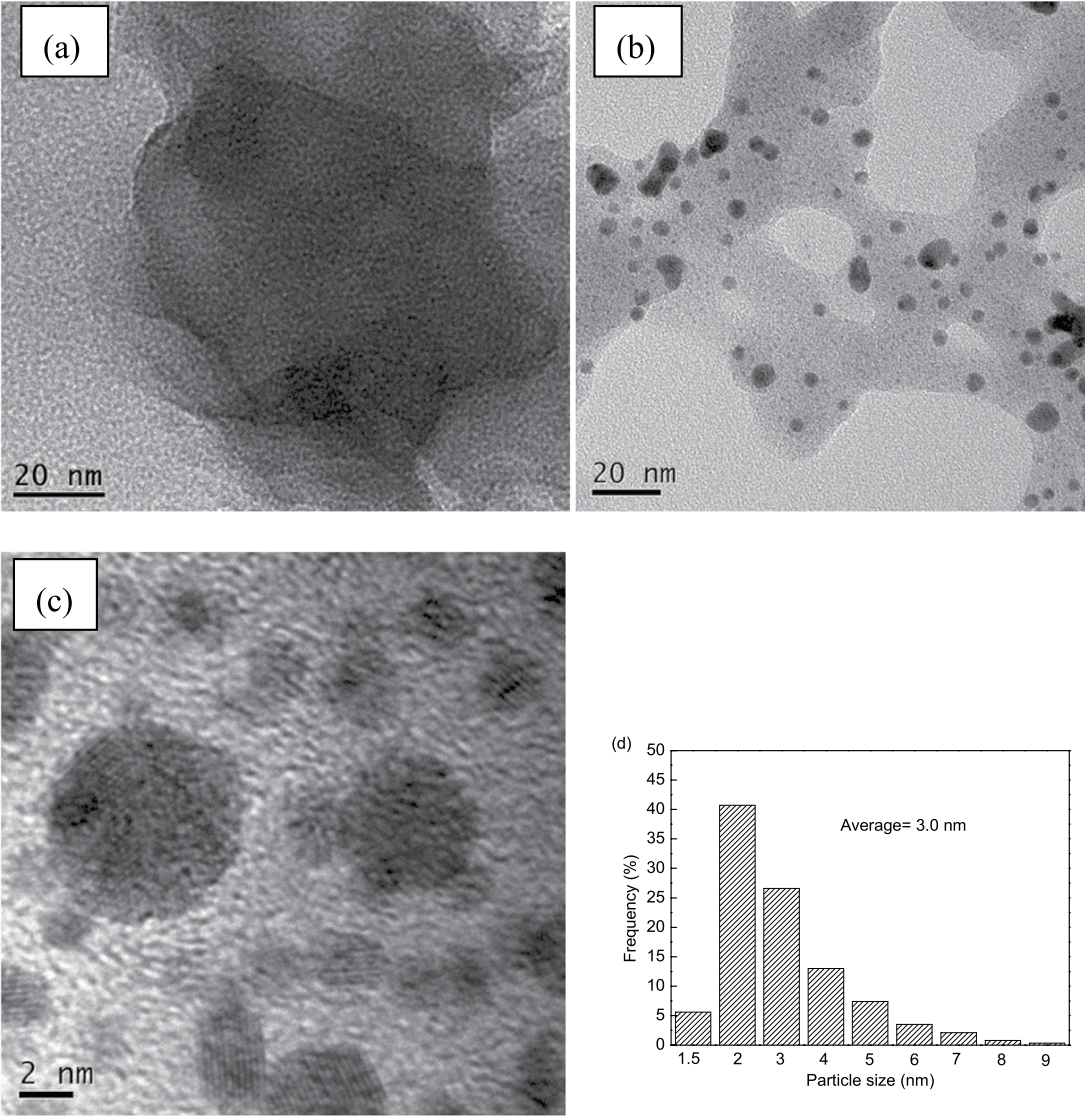
TEM image of as-synthesized IRMOF-3-LA-Au (**a**) and recovered IRMOF-3-LA-Au after four cycles of A^3^ coupling reactions at 80 °C (**b** and **c**), and the corresponding size distribution plot of Au nanoparticles (**d**).

Figure 3 shows the TEM images of the as-synthesized IRMOF-3-LA-Au and recovered IRMOF-3-LA-Au after four cycles of A^3^ coupling reactions at 80 °C. Au° nanoparticles were not observed on the as-synthesized IRMOF-3-LA-Au, which is consistent with the XRD patterns of as-synthesized IRMOF-3-LA-Au. However, Au° nanoparticles with an average particle size of 3.0 nm were visible on the recovered IRMOF-3-LA-Au. This result is consistent with the H_2_-TPR results.

Inductively coupled plasma atomic emission spectroscopy (ICP-AES) analysis indicated that the Au content of IRMOF-3-LA-Au was 4.9 wt% (0.2487 mmol/g), corresponding to 6.8% –NH_2_ groups of IRMOF-3 having been functionalized. The loading of Au in recovered IRMOF-3-LA-Au after the four cycles of A^3^ coupling reactions at 80 °C was 4.1 wt% measured by ICP-AES. A large leaching of gold (ca. 16.3%) over the recovered IRMOF-3-LA-Au was observed as evidenced by ICP-AES.

### Catalytic Tests

**Figure 4:**
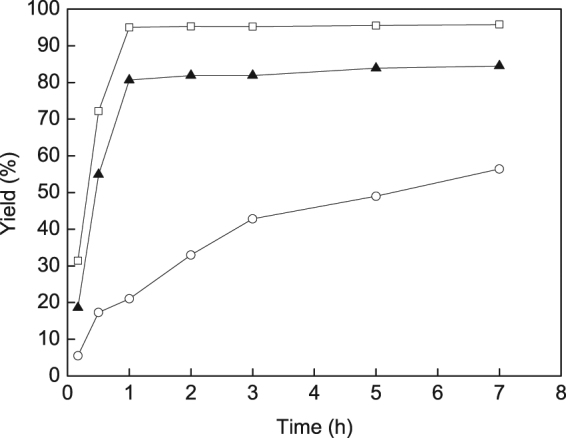
Comparison of catalytic activity for A^3^ coupling reaction of benzaldehyde, phenylacetylene, and piperidine with 1,4-dioxane as solvent on IRMOF-3-LA-Au at 80 °C (open circle), 100 °C (triangle), and 120 °C (open square).

The prepared Au^3+^–containing catalysts with Au loading of 4.9 wt% on the MOF of IRMOF-3 were tested in the A^3^ coupling reaction. The reaction of benzaldehyde, phenylacetylene, and piperidine in a 1:1.2:1.3 mole ratio with 1,4-dioxane as solvent was selected as the model reaction. Given that temperature plays a crucial role in catalysis, the reaction was performed under varying reaction temperatures. Figure 4 shows the catalytic activity of IRMOF-3-LA-Au in the A^3^ coupling reaction of benzaldehyde, phenylacetylene, and piperidine at 80 °C, 100 °C, and 120 °C with 1,4-dioxane as solvent. IRMOF-3-LA-Au exhibited good catalytic activity in the A^3^ coupling reaction of benzaldehyde, phenylacetylene, and piperidine. The yield of propargylamines was 56% within 7 h at 80 °C. As temperature increased from 80 °C to 100 °C, the yield of propargylamines increased to 81% within 1 h. Further increase in temperature to 120 °C, a yield of 95% was obtained within 1 h. If one calculates the turnover number (TON) from the maximum yield taking into account the total Au content of the catalyst, the values obtained are 8, 12, and 14 for IRMOF-3-LA-Au at 80 °C, 100 °C, and 120 °C, respectively. In our previous work, maximum benzaldehyde conversion of 77% were obtained within 0.5 h at 120 °C over 4.6%Au/IRMOF-3 containing coexisting Au^3+^ ions and Au^0^ nanoparticles (Au^3+^/Au^0^ = 0.2)^17^. Au^0^ nanoparticles supported on IRMOF-3 yielded the desired propargylamines with a benzaldehyde conversion of 8% and 16% at 120 °C for 5 h over 0.6%Au/IRMOF-3 and 3.2%Au/IRMOF-3, respectively^17^. Taking into account the total Au content of the catalyst, the TON numbers from the maximum conversions were calculated to be 12, 9, and 4 for 4.6%Au/IRMOF-3, 0.6%Au/IRMOF-3, and 3.2%Au/IRMOF-3, respectively. Copper nanoparticles supported on starch micro particles (CuNPs@MS) gave a yield of 30% within 20 h at 60 °C for the A^3^ coupling reaction of benzaldehyde, phenylacetylene, and piperidine in 1,4-dioxane with a TON of 100 based on the total Cu content^59^. NiO nanoparticles gave a yield of 95% at 120 °C for 22 h under N_2_ atmosphere in toluene with a TON number of 6^60^. Based on the above results, although the TON number of IRMOF-3-LA-Au is still less than those of CuNPs@MS, the catalyst of IRMOF-3-LA-Au shows higher activity than 4.6%Au/IRMOF-3, 0.6%Au/IRMOF-3, 3.2%Au/IRMOF-3, and NiO nanoparticles.

To demonstrate the generality of IRMOF-3-LA-Au as catalyst, the A^3^ coupling of different combinations of aldehydes, alkynes, and amines were tested. First, we extended the reaction to various substituted benzaldehydes bearing electron-donating or electron-withdrawing groups (Table 1, entries 1–6). The coupling reaction with benzaldehyde bearing electron-donating groups, such as –CH_3_ and –CH_3_O, displayed higher yields than the benzaldehyde bearing electron-withdrawing groups (Table 1, entries 1–6). In addition, reactions with aliphatic aldehydes, such as cyclohexanecarboxaldehyde, *n*-octyl aldehyde, and *n*-heptyl aldehyde also displayed good yield of propargylamines (Table 1, entries 7–9). Moreover, we attempted to expand the reaction to other alkynes, such as alkyl- and aryl-substituted terminal alkynes (Table 1, entries 10–18). The aromatic alkynes were also suitable substrates for A^3^ coupling reaction. The yield of propargylamines decreased with increasing the chain length of straight-chain alkyl substituted phenylacetylene (Table 1, entries 10–15). (Trimethylsilyl)acetylene also afforded propargylamines in good yields (Table 1, entries 16, 17). The yields of propargylamines reached to 72% and 99% at 80 °C and 100 °C, respectively. By contrast, 1-octyne afforded a 25% yield of propargylamines at 100 °C within 1 h. Furthermore, we investigated the scope of amines, including alicyclic and dialkyl amines (Table 1, entries 19–21). Reactions with alicyclic amines such as morpholine and pyrrolidine proceeded efficiently and resulted in moderate yield. However, the reaction proceeded slowly in the presence of dialkyl amine such as diethylamine with benzaldehyde and phenylacetylene to afford product in 28% yield.

**Table 1:** IRMOF-3-LA-Au catalyzed three-component coupling of aldehyde, alkyne and amine^a^.

Entry	R^1^	R^2^	R^3^R^4^NH	Temp./°C	Time/h	Yield/%
1	3-ClC_6_H_4_	Ph	piperidine	80	7	35
2	3-ClC_6_H_4_	Ph	piperidine	100	1	65
3	4-MeC_6_H_4_	Ph	piperidine	80	7	54
4	4-MeC_6_H_4_	Ph	piperidine	100	1	79
5	4-MeOC_6_H_4_	Ph	piperidine	80	7	72
6	4-MeOC_6_H_4_	Ph	piperidine	100	1	91
7	Cyclohexyl	Ph	piperidine	80	7	93
8	Heptyl	Ph	piperidine	80	7	94
9	Hexyl	Ph	piperidine	80	7	99
10	Ph	4-MeC_6_H_4_	piperidine	80	7	65
11	Ph	4-MeC_6_H_4_	piperidine	100	1	79
12	Ph	4-EtC_6_H_4_	piperidine	80	7	54
13	Ph	4-EtC_6_H_4_	piperidine	100	1	66
14	Ph	4-ButC_6_H_4_	piperidine	80	7	31
15	Ph	4-ButC_6_H_4_	piperidine	100	1	55
16	Ph	(CH_3_)_3_Si	piperidine	80	7	72
17	Ph	(CH_3_)_3_Si	piperidine	100	1	99
18	Ph	Hexyl	piperidine	100	1	25
19	Ph	Ph	morpholine	100	1	67
20	Ph	Ph	pyrrolidine	100	1	64
21	Ph	Ph	diethylamine	100	1	28

^a^ , Reaction conditions: aldehyde (0.25 mmol), amine (0.30 mmol), alkyne (0.33 mmol), IRMOF-3-LA-Au (0.07 g).

The reusability studies of IRMOF-3-LA-Au were carried out on the A^3^ coupling reaction of benzaldehyde, phenylacetylene, and piperidine in 1,4-dioxane at 80 °C. The catalyst was recovered by separating IRMOF-3-LA-Au from the liquid component of the mixture via extensive centrifugation. The recovered catalyst was washed twice with 1,4-dioxane, dried at 50 °C under vaccum for 1 h, and reused. In four successive cycles, yields were 56%, 48%, 35%, and 26% at 80 °C for 7 h. The Au^3+^ species on the recovered IRMOF-3-LA-Au were reduced into Au^0^ during A^3^ coupling reaction as revealed by XRD, H_2_-TPR and TEM results. The recovered IRMOF-3-LA-Au contains coexisting active centers: Au^3+^ ions and Au^0^ nanoparticles. Studies have reported that the activity of Au-based catalysts in A^3^ coupling reaction decreases in the following order: Au^3+^ > Au^0^ ^51,61^. Therefore, reduction of Au^3+^ species is possibly one of the main reasons for the deactivation of IRMOF-3-LA-Au catalysts.

We performed a leaching test to examine if there are any homogeneous active species in solution that could catalyze the A^3^ coupling reaction^62–66^. The hot filtration test was carried out by stopping the A^3^ coupling reaction of benzaldehyde, phenylacetylene, and piperidine after 2 h at 80 °C. The yield of propargylamines reached to 33% within 2 h at 80 °C. The reaction solution was removed quickly from the catalyst of IRMOF-3-LA-Au and was transferred to another reactor under the same reaction conditions. The yield of propargylamines increased to 37% after reaction another 2 h at 80 °C. These results demonstrate that there are some leaching active species under the reaction conditions. This result was confirmed by ICP-AES analysis of the recovered IRMOF-3-LA-Au after four reaction cycles which showed large leaching of Au species (16.3%) to reaction medium.

## Conclusions

We successfully designed a new heterogeneous catalyst for A^3^ coupling reactions by anchoring Au^3+^ onto microporous IRMOF-3 via a postsynthetic modification method. IRMOF-3-LA-Au showed high catalytic activity for the A^3^ coupling reaction of aldehyde, alkyne, and amine in 1,4-dioxane. In addition, IRMOF-3-LA-Au is applicable for a wide range of substrates, including aromatic and aliphatic aldehydes, alkyl- and aryl-substituted terminal alkynes, and alicyclic amines. IRMOF-3-LA-Au can be easily separated from the mixture and can be reused for four cycles, thus making the catalyst highly desirable in addressing environmental concerns.

## Methods

### IRMOF-3 Preparation

IRMOF-3 was synthesized using a solvothermal method. In brief, 2-aminoterephthalic acid (NH_2_-BDC, 0.3723 g, 2 mmol) and Zn(NO_3_)_2_·6H_2_O (1.78 g, 7 mmol) were dissolved in 70 mL DMF and stirred for 30 min at room temperature. The mixture was subsequently transferred into a 100-milliliter Teflon Liner with a metallic Paar Bomb. The container was placed in an oven at 100 °C for 24 h to yield brown crystals. After cooling overnight, the mother liquor was decanted and the as-synthesized materials were washed with 3 × 20 mL of DMF and then with 3 × 20 mL of chloroform (CHCl_3_). The product was dried at 50 °C for 12 h under vacuum before use^17,67,68^.

### IRMOF-3-LA Preparation

In a typical synthesis, freshly dried IRMOF-3 (0.3 g, 1.095 mmol eq of –NH_2_) was dispersed in a 25 mL vial containing 10 mL of acetonitrile (CH_3_CN). Lactic acid (3.83 mmol, 345.0 mg) was added with stirring (400 r/min), and the mixture was stirred under reflux for 3 h. The sample was collected by centrifugation and washed three times with CH_3_CN (3 × 8 mL). The solid was then dried at 50 °C for 4 h under vacuum to yield the final compound, IRMOF-3-LA.

### IRMOF-3-LA-Au Preparation

Gold functionalized IRMOF-3 was prepared via postsynthetic modification. HAuCl_4_·4H_2_O (0.052 g) was dissolved in CH_3_CN (0.50 mL). HAuCl_4_·4H_2_O solution was subsequently added dropwise into the dried IRMOF-3 (0.50 g) at room temperature. The sample was stored overnight and dried at 50 °C for 3 h under vacuum.

### Catalyst Characterization

XRD was performed with Brüker D8 diffractometer operated with a Cu K*α* radiation (λ = 1.541 Å) at 40 mA and 40 kV. Nitrogen physisorption measurements were carried out using a Quantachrome instrument at 77 K. Samples weighing 80–100 mg were outgassed for 12 h at 50 °C prior to measurement. FT-IR spectrum (400–4000 cm^−1^) was recorded from KBr pellet in NICOLET 5700 FT-IR spectrometer. ^1^H-NMR spectra were recorded on Brüker Advance III HD spectrometer (500 MHz). Approximately 5 mg of IRMOF-3 and IRMOF-3-LA samples were dried under vacuum at 90 °C for 12 h and digested with sonication in 500 μL DMSO-d_6_ and 100 μL dilute DCl (23 μL of 35% DCl in D_2_O diluted with 1.0 mL of DMSO-d_6_). Approximately 10–20 mg of IRMOF-3, IRMOF-3-LA, or IRMOF-3-LA-Au was used for TGA measurements. Samples were analyzed under a stream of N_2_ using a TA SDT Q600 running from room temperature to 700 °C with a scan rate of 15 °C/min. ICP-AES analysis was performed on a Perkin–Elmer Optima 7000 DV apparatus. TEM studies were carried out with a FEI TECNAI F20 Transmission Electron Microscope. H_2_-TPR was conducted on a ChemStar apparatus equipped with a TCD detector. A 50-milligram portion of each sample was loaded in a U-shaped quartz microreactor and heated at a ramping rate of 10°C/min in 5 vol% H_2_ in Ar (a total flow of 50 mL/min).

### Catalytic Measurements

A mixture of IRMOF-3-LA-Au (70 mg, 0.0177 mmol Au), aldehyde (0.25 mmol), alkyne (0.33 mmol), amine (0.30 mmol), and 1,4-dioxane (1.5 mL) was placed in a closed glass reactor (8.0 mL, SYNTHWARE) and vigorously stirred at 80 °C–120 °C in an oil bath for 0.15–7 h. After the reaction was completed, the mixture was cooled to room temperature and centrifuged at 14000 rpm for 6 min. IRMOF-3-LA-Au was deposited into the bottom of a centrifuge tube and separated from the liquid component of the mixture. The liquid was analyzed using a GC (GC-1100, capillary column, SE-54).

## Electronic supplementary material

Supporting information
