# Supplementary material for: Anchorage of Au3+ into Modified Isoreticular Metal–Organic Framework-3 as a Heterogeneous Catalyst for the Synthesis of Propargylamines
Source: Sci Rep. 2017 Oct 5;7:12709. doi: 10.1038/s41598-017-13081-0 (PMC5629214; doi:10.1038/s41598-017-13081-0)
Supplement: Supplementary file 1 — Supporting information [file 41598_2017_13081_MOESM1_ESM.doc]

Supporting information to

**Anchorage of Au^3+^ into Modified Isoreticular Metal–Organic Framework-3 as** **a Heterogeneous Catalyst for the Synthesis of** **Propargylamines**

**Lili Liu, Xishi Tai, Xiaojing Zhou, Chunling Xin & Yongmei Yan**

School of Chemistry & Chemical Engineering and Environmental Engineering, Weifang University, Weifang 261061, China;

Correspondence and requests for materials should be addressed to L.L. (email: [liulili122@wfu.edu.cn](mailto:liulili122@wfu.edu.cn)) and X.T. (email: [taixs@wfu.edu.cn](mailto:taixs@wfu.edu.cn))

Figure Caption:

Figure S1. N_2_ adsorption–desorption isotherms of IRMOF-3 (a), IRMOF-3-LA-Au (b), and recovered IRMOF-3-LA-Au (c).

Figure S2. Liquid-state ^1^H-NMR spectra of IRMOF-3 (a) and IRMOF-3-LA (b)

Figure S3. TG-DTA curves of IRMOF-3 (a), IRMOF-3-LA (b), and IRMOF-3-LA-Au (c)

**Figure S1**. N_2_ adsorption–desorption isotherms of IRMOF-3 (a), IRMOF-3-LA-Au (b), and recovered IRMOF-3-LA-Au after four cycles of A^3^ coupling reactions at 80 °C (c);

(a)

(b)

**Figure S2.** Liquid-state ^1^H-NMR spectra of IRMOF-3 (a) and IRMOF-3-LA (b)

**Figure S3**. TG-DTA curves of IRMOF-3 (a), IRMOF-3-LA (b), and IRMOF-3-LA-Au (c).
